# Effect of fungicides on epiphytic yeasts associated with strawberry

**DOI:** 10.1002/mbo3.85

**Published:** 2013-04-04

**Authors:** Jane Debode, Wendy Van Hemelrijck, Piet Creemers, Martine Maes

**Affiliations:** 1 Plant Sciences Unit – Crop Protection, Institute for Agricultural and Fisheries Research (ILVO)Burg. van Gansberghelaan 96 bus 2, 9820, Merelbeke, Belgium; 2 Research centre for fruit cultivation (pcfruit vzw)Fruittuinweg 1, 3800, Sint-Truiden, Belgium

**Keywords:** DGGE, *Fragaria × ananassa*, fungicide, fungicide resistance, phylloplane, plating, yeast

## Abstract

We studied the effect of two commonly used fungicides on the epiphytic yeast community of strawberry. Greenhouse and field experiments were conducted applying Switch (cyprodinil plus fludioxonil) or Signum (boscalid plus pyraclostrobin) to strawberry plants. Yeasts on leaves and fruits were assessed on treated and untreated plants at several time points via plating and denaturing gradient gel electrophoresis (DGGE) analysis. The yeast counts on plates of the treated plants were similar to the control plants. Unripe fruits had 10 times larger yeast concentrations than ripe fruits or leaves. Some dominant yeast types were isolated and in vitro tests showed that they were at least 10 times less sensitive to Switch and Signum as compared with two important fungal strawberry pathogens *Botrytis cinerea* and *Colletotrichum acutatum*, which are the targets for the fungicide control. DGGE analysis showed that the applied fungicides had no effect on the composition of the yeast communities, while the growing system, strawberry tissue, and sampling time did affect the yeast communities. The yeast species most commonly identified were *Cryptococcus, Rhodotorula*, and *Sporobolomyces*. These results point toward the potential applicability of natural occurring yeast antagonists into an integrated disease control strategy for strawberry diseases.

## Introduction

Strawberry (*Fragaria* × *ananassa*) is one of the most widely grown small fruit crops in the world. Fungal diseases of strawberry, mainly caused by *Colletotrichum acutatum* and *Botrytis cinerea*, are responsible for severe economic losses (Maas 1998; Wedge et al. 2007).

*Colletotrichum acutatum* mainly causes anthracnose fruit rot. Management of this disease is greatly hindered by the difficulty in detection and control of this fungus during symptomless infections on strawberry leaves and unripe fruit (Debode et al. 2009; Van Hemelrijck et al. 2010; Guidarelli et al. 2011). *Botrytis cinerea* causes gray mold of strawberry. Although fruit rot results mainly from symptomless infections of flower parts and develops once fruit begins to ripen (Powelson 1960; Jarvis and Borecka 1968; Bristow et al. 1986), infection of leaves by *B. cinerea* may lead to increased inoculum production when leaves are senescing in a perennial growing system (Braun and Sutton 1988). Currently, the control of fungal diseases of strawberry relies mainly on the use of fungicides. However, consumers are demanding that chemical residues on fruits be reduced, and many fungi are developing resistance to widely used synthetic fungicides (Wedge et al. 2007). Control strategies which reduce fungicide use must therefore be developed.

Biological control of strawberry fungal diseases using yeasts has been proposed as a promising alternative, either alone or as part of integrated pest management (e.g., Guetsky et al. 2002; Wszelaki and Mitcham 2003; Zhang et al. 2007). Yeasts are suitable for biocontrol, as they generally do not produce allergenic spores, mycotoxins, or antibiotics, in contrast to some fungal or bacterial antagonists (Droby and Chalutz 1994). Moreover, yeasts are known to efficiently colonize the epiphytic environment, which could antagonize the introduction and development of plant pathogens (Buck and Burpee 2002; Fonseca and Inácio 2006).

But before epiphytic yeasts can be fully tested for exploitation as protective and biological control organisms, their behavior under current plant growth conditions (e.g., in the greenhouse or field) must be studied, including their reaction to the application of fungicides (Buck and Burpee 2002). Studies investigating such effects have been done mainly on grapes, turf-type grasses, and apples, and the results obtained are mainly based on culture-based analysis of the yeast communities (Hislop and Cox 1969; Andrews and Kenerley 1978; Teixido et al. 1999; Buck and Burpee 2002; Comitini and Ciani 2008; Cadez et al. 2010). These traditional plating techniques make it difficult to detect qualitative changes and relative dominance in epiphytic yeast communities (Buck and Burpee 2002). Molecular analytical methods based on polymerase chain reaction (PCR) Denaturing Gradient Gel Electrophoresis (DGGE) have been proposed as a tool to study the diversity and dynamics of epiphytic yeast on grapes (Prakitchaiwattana et al. 2004). During that study, total DNA was extracted from grape rinses and subjected to PCR using universal primers that target the D1/D2 domain of the 26S rDNA.

Very little is known about the yeast community of strawberry. Additionally, it is unknown what effect fungicides used for fruit rot control have on the yeast communities on strawberry. The objectives of this study were (i) to assess the effect of two commonly used strawberry fruit rot fungicides on the density and diversity of epiphytic yeast on greenhouse- and field-grown strawberry using traditional plating and DGGE analysis, (ii) to determine the in vitro fungicide sensitivity of yeasts as compared with fruit rot fungi, (iii) to study the yeast ecology of greenhouse- and field-grown strawberry fruit and leaves at different time points using traditional plating and DGGE analysis, and (iv) to identify the dominant epiphytic yeast species from plates and DGGE using partial rDNA sequencing. This line of research fits within our long-term goal of incorporating naturally occurring yeast antagonists into an integrated disease control strategy for strawberry.

## Materials and Methods

### Greenhouse experiment

Assessment of the effect of a fungicide application on epiphytic yeast communities on strawberry (cv. Elsanta) was first conducted in a greenhouse experiment during the summer of 2009 at the Institute for Agricultural and Fisheries Research (ILVO), Merelbeke, Belgium. Forty strawberry transplants were planted in individual pots and placed in a greenhouse at 18–28°C and a 16-h day/light regime. Four weeks after planting (Time 0), one fully expanded leaf from each plant was excised at the petiole base and pooled into four samples of 10 leaves each. Subsequently, 20 of the 40 plants were sprayed with Switch (cyprodinil + fludioxonil WG; 1 g/L) and 20 plants were sprayed with water (nontreated control) until runoff using a hand trigger sprayer. Spray treatments were repeated 6 days later. One day after the second treatment (Time 1), fully expanded leaves were sampled, pooled per treatment as described above, and analyzed. Yeasts were isolated from the leaves by placing the leaf samples in a flask with 1 L 0.1% Tween 80 solution and shaking for 30 min in day light at 150 rpm. Subsequently, the rinse was poured from the leaves and examined further by plating and PCR-DGGE analysis (see below).

### Field experiment

After conducting the small greenhouse experiment, a more extended experiment was done in the field at Proefcentrum Fruitteelt (pcfruit) vzw, a research station in Sint-Truiden, Belgium. Cold-stored transplants were planted in May 2009 and a normal strawberry production season followed. At the end of this production period (autumn 2009), plants were mowed. In May 2010, the field experiment was designed to assess the effect of a fixed schedule spraying (1× per week) of two fungicides on epiphytic yeast communities on (un)ripe strawberry fruits and leaves (cv. Elsanta). Treatment plots were arranged as a randomized complete block with four replicates. Each replicate plot measured 5 × 1 m and contained approximately 30 plants. Plots were either not treated or treated with either Signum (pyraclostrobin + boscalid WG; 1.8 kg/ha) or Switch (cyprodinil + fludioxonil WG; 1.0 kg/ha) using a Knapsack sprayer (type Stihl, model SR430; Puurs, Belgium). The fungicides were applied weekly starting from the 5th of May until the 2nd of June, resulting in five spray applications. Untreated plots were labeled 1–4, Switch-treated plots 5–8, and Signum-treated plots 9–12. Samples of strawberry leaves and (un)ripe fruits were collected from each plot at three time points (Time 0 = 4th of May, Time 1 = 24th of June, and Time 2 = 30th of June), always at the beginning of the day. Each sample consisted of 10 (un)ripe fruits or fully expanded leaves taken randomly from each plot. Yeasts were isolated from the leaves or fruit by placing the samples in a flask in 1 L 0.1% Tween 80 solution and shaking for 30 min in day light at 150 rpm. Subsequently, the rinse was poured from the leaves or fruit and examined further by plating and PCR-DGGE analysis (see below).

At harvest, natural *B. cinerea* and *C. acutatum* infections were recorded by counting the total number of fruits showing gray mold rot (*B. cinerea*) and anthracnose fruit rot (*C. acutatum*).

### Plating and rDNA sequence-based identification of yeast isolates

Ten microliter of each rinse sample and of a 10-fold dilution were plated on potato dextrose agar (PDA) plates. The 10 μL drops were spread over the entire plate by rubbing the plate with a glass rod and this was replicated once. Plates were incubated at 25°C for 4 days, after which representatives of the different types of yeast colonies were recognized using a microscope and a yeast morphology flow chart (http://www.doctorfungus.org/thelabor/sec12.pdf, access date: 28 September 2012). Yeast colonies were counted (only for the field experiment) and colonies that differed in color, shape, or texture were purified by restreaking on PDA and incubating the plates at 20°C in the dark.

Subsequently, DNA was extracted from each yeast isolate using the PUREGENE kit (Qiagen, Belgium) following the manufacturer's instructions. The yeasts were identified by sequence analysis of the D1/D2 domain of the 26S rDNA. This region was PCR amplified using primers NL1 and NL4 (Kurtzman and Robnett 1997), and the fragment (∼600 bp long) was separated on agarose gel and purified using the Nucleospin Extract II Kit (Macherey-Nagel, Germany). Bidirectional sequencing was done by Macrogen (South Korea). The sequences were compared using BLAST with sequences available in the NCBI GenBank database. Unique sequences were submitted to that database (JN636804-JN636813).

### PCR-DGGE and sequencing of the dominant DGGE bands

Four hundred milliliter of each rinse sample was centrifuged at 16,000 *g* for 15 min at 4°C. The cell pellets were stored at −20°C until DNA extraction using the PUREGENE kit following the manufacturer's instructions. DNA extracted from these pellets and from pure yeast isolates (as outline above) was amplified using the 26S rDNA-based nested PCR protocol described by Prakitchaiwattana et al. (2004). Specifically, the first PCR was conducted with the forward primer NL1 and reverse primer NL4. Amplification was done in a standard 50-μL reaction mixture containing 36.6 μL water, 5.0 μL 10× buffer with MgCl_2_ (Roche, Germany), 0.4 U of FastStartTaq polymerase (Roche), each deoxynucleotide triphosphate at a concentration of 0.2 mmol/L, each primer at a concentration of 0.2 μmol/L, and the DNA template at a final concentration of 10 ng. PCR was run for 36 cycles with annealing at 52°C, extension at 72°C for 2 min, and denaturation at 94°C for 1 min. The amplicon (∼600 bp) from the first PCR was diluted and further amplified with a second PCR using the GC-clamp primer NL1 and the forward primer LS2. The conditions of this reaction were the same as those described for the first PCR. The GC-clamped PCR products (∼250 bp long) were purified by ethanol precipitation and resuspended in 10 μL water. DNA was then quantified using a Nanodrop spectrophotometer (Isogen Life Sciences, the Netherlands), diluted to 300 ng/μL (for DNA from the leaf and fruit samples) or 100 ng/μL (for DNA from the pure yeast isolates), and stored at −20°C until needed. As a ladder for standardization of the DGGE gel runs, 16S rDNA DGGE-PCR products from different bacterial species were pooled. Five microliter of PCR product was run in DGGE on a DcodeTM Detection system (Bio-Rad, Belgium). The gels had 8% (w/v) polyacrylamide (polyacrylamide:bisacrylamide, 37.5:1) and a denaturing gradient from 30% to 60% (v/v) of urea and formamide (Prakitchaiwattana et al. 2004). Electrophoresis was performed at a constant voltage of 50 V for 17 h with a constant temperature of 60°C. After electrophoresis, the gels were stained in 1 × TAE buffer pH 8, containing 1 × SYBR Gold solution (Molecular Probes/Invitrogen, Belgium) during 20 min with slight shaking (150 rpm) and photographed under UV. The DGGE profiles of the different samples were compared using band-based comparison using Jaccard's UPGMA clustering tool within BIONUMERICS (version 5.1, Applied Maths, Belgium).

Dominant DGGE bands were carefully excised from the gels using sterile razor blades. The gel pieces were soaked for 10 min in 50-μL PCR mix, containing primers NL1 and LS2, buffer, Taq DNA polymerase, and water. After removal of the gel debris by pipetting the liquid into a new tube, the eluted DNA was reamplified by PCR. Subsequently, the PCR products were purified on agarose gel and the fragment (∼250 bp) was extracted using the Nucleospin Extract II Kit. Cloning of the purified PCR product was done in a pCRII-TOPO vector with the TOPO TA Cloning 151 Kit (Invitrogen, Belgium). DNA of the plasmid clones was extracted using a NucleoSpin plasmid DNA purification kit (Macherey-Nagel). The plasmid inserts were bidirectionally sequenced by Macrogen using the plasmid primers Sp6 and T7. The obtained sequences were aligned using the multiple alignment tool of BIONUMERICS, compared with the GenBank database of NCBI using BLAST, and entered in the same database when unique sequences were identified (JN636797-JN636803).

### In vitro fungicide sensitivity of yeasts and fruit rot fungi

In vitro sensitivity of epiphytic yeast isolates and isolates of the fruit rot fungi *C. acutatum* and *B. cinerea* to Switch and Signum was evaluated on fungicide-amended PDA plates. The yeast isolates were selected from the greenhouse and field experiment (Table 1). The *C. acutatum* and the *B. cinerea* isolates were PCF192, PCF229, and PCF714 (Van Hemelrijck et al. 2010) and PCF232 and PCF895 (this study), respectively. Cell (yeasts) and conidial (fruit rot fungi) suspensions were prepared by flooding fully grown PDA cultures (14 days old) with sterile water and rubbing the surface with a glass rod. The fruit rot fungi suspensions were filtered through autoclaved cheesecloth to remove the mycelia. The cell and spore concentrations were determined using a hemocytometer and each isolate was suspended in sterile distilled water at ∼1 × 10^3^ cells (yeast) or conidia (fruit rot fungi) per mL. To determine relative sensitivity of the yeasts and fruit rot fungi to Switch and Signum, one 100 μL aliquot of each suspension was applied to petri dishes containing PDA amended with 100 mg/L chloramphenicol and different concentrations of each fungicide. Commercial formulations of the fungicide were diluted in cooled PDA and poured into petri dishes. Concentrations of Swith and Signum were 0.01, 0.1, 1, 10, 100, and 1000 ppm. Dishes of nonamended PDA served as controls. The plates were incubated in the dark at 20°C. Yeast and fungal growth on the plates was evaluated 3 days after incubation by counting the colonies that developed from the cells/spores. Data were pooled as per category (*C. acutatum*, *B. cinerea*, and epiphytic yeasts).

**Table 1:** Tentative phylogenetic affiliation, based on 26S rDNA sequencing, of the dominant epiphytic yeast types isolated by plating from the surface of strawberry leaf and fruit tissues

Strain^1^	Genbank accession #	Experiment location	Strawberry tissue	Closest sequence relatives						
				Strain(s)	Genbank accession #	Homology %
G1	JN636809	greenhouse	leaf	*Candida* sp. GA1S07	FJ527144	98
G2*	JN636810	greenhouse	leaf	*Cryptococcus* sp. VTT C-04547	DQ377668	100
G3	JN636811	greenhouse	leaf	*Sakaguchia dacryoidea* CBS 6353	AF189973	94
G4	JN636812	greenhouse	leaf	*Cryptococcus* sp. KCTC 17100	AF459675	100
G5*	JN636813	greenhouse	leaf	*Sporobolomyces* sp. KCTC 17098	AF459715	95
F1*	JN636806	field	leaf	*Cryptococcus* sp. HB 982	FM242574	99
F2	JN636807	field	leaf	*Cryptococcus heveanensis* YFL1.1	HQ629577	99
F3	JN636808	field	ripe fruit	*Rhodotorula graminis* WP1	EU563930	99
F4*	JN636804	field	unripe fruit	*Pichia guilliermondii* SYHZS-1	EU809436	100
F5*	JN636805	field	unripe fruit	*Metschnikowia pulcherrima* Afen	EU272042	95

^1^Isolates, representative of strain followed by ^*^ were used for in vitro fungicide sensitivity tests.

### Statistical analysis

Statistical analysis of yeast density data was done using STATISTICA (Statsoft, Tulsa, OK). Data were analyzed using analysis of variance (ANOVA), and means of different treatments were compared using Fisher's LSD multiple range test (*P* < 0.05).

## Results

### Greenhouse experiment

**Figure 1:**
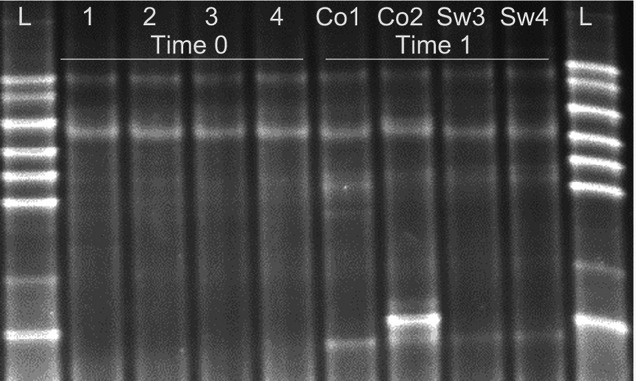
Greenhouse experiment. Denaturing gradient gel electrophoresis (DGGE) profile of yeasts on the surface of strawberry leaf tissue sampled before (Time 0) and after (Time 1) Switch (Sw) treatment. Each lane represents a composite sample of 10 leaves from 10 different plants. Water-treated plants served as control (Co). L = DGGE ladder.

Fungi, bacteria, and yeasts present in leaf washes were detected on the PDA plates. Colonies representing unique color and morphological characteristics from each group, were isolated, purified, and identified based on their partial 26S rDNA sequences (∼600 bp long). Five dominant yeast types were identified (G1–G5, Table 1). Four yeasts belonged to the basidiomycetes, represented by creamy or lightly pigmented *Cryptococcus* species and pink- to red-pigmented species of *Sakaguchia* and *Sporidiobolus*, and one belonged to the ascomycete species *Candida*, represented by a white to creamy colony type. Similar dominant yeast colony types were present on the PDA plates regardless of treatment. DGGE profiles of the epiphytic yeast communities were not consistently different between fungicide-treated and nontreated plants, but cluster analysis elucidated a profile shift between the two sampling times (Fig. 1, clustering data not presented). Specifically, two dominant bands were detected in all lanes at the two time points, but at Time 1 additional band areas were more pronounced both in the control and fungicide-treated samples, as compared with the profiles at Time 0. This indicates that the yeast flora on the leaves had changed in time. In addition, at Time 1, the control sample Co2 was the most aberrant from the three other samples, which were similar. None of the DGGE bands in the profiles corresponded to the DGGE bands produced by the five strains isolated by plating (G1–G5) (data not presented).

### Field experiment

In the control plots, 2.0% of the harvested fruits were affected with *B. cinerea* and 0.3% with *C. acutatum*. In the fungicide plots, 0.2% was affected with *B. cinerea* and 0.0% with *C. acutatum*.

For the plants grown in the field, the yeasts isolated on plate and identified by 26S rDNA sequencing belonged to basidiomycetes white-yellow *Cryptococcus* and pink- to red-pigmented *Rhodotorula*. In addition, two ascomycete yeasts were identified: *Pichia* and *Metschnikowia*, represented by creamy-to-yellow colonies (Table 1). Similar dominant yeast colony types were isolated from fungicide-treated and nontreated samples.

Significantly greater but variable concentrations of adherent yeasts were counted on the unripe fruit (Time 1) as compared with the other strawberry tissues sampled at Times 0 and 2 (Table 2). In addition, there was a 97% reduction in the amount of yeasts counted on the leaves at Time 2 (after spraying) as compared with Time 0 (before spraying). Because of the sampling set × treatment interaction, each sampling set was analyzed separately. For each sampling set, no significant difference in total yeast communities on fungicide-treated plant samples versus untreated plant samples was found (Table 2).

**Table 2:** Mean yeast concentrations (CFU/100 μL tissue rinse) per plot isolated from either strawberry leaf or fruit tissues determined by culture plating onto potato dextrose agar (2 replicate plates per plot)

	Untreated leaves Time 0	Fungicide sprayings	Treatment	Unripe fruit Time 1	Ripe fruit Time 2	Leaves Time 2
	
	Dates					
	
Plot	04 May 2010	5-12-19-27 May and 02 June 2010		24 June 2010	30 June 2010	30 June 2010
1	1.0		Untreated	10.0	2.0	0.0
2	0.0		Untreated	10.0	0.0	0.0
3	1.0		Untreated	30.0	0.0	0.0
4	2.5		Untreated	135.0	47.0	0.0
Mean	1.1a		Untreated	46.0a	12.3a	0.0a
5	5.0		Switch	130.0	5.0	0.5
6	1.0		Switch	85.0	1.5	0.0
7	1.5		Switch	25.0	0.0	0.0
8	2.0		Switch	80.0	0.0	0.0
Mean	2.4b		Switch	80.0a	1.6a	0.1a
9	3.0		Signum	5.0	0.5	0.0
10	6.5		Signum	20.0	0.0	0.0
11	6.0		Signum	20.0	0.5	0.0
12	12.0		Signum	100.0	0.0	1.0
Mean	6.9b		Signum	36.3a	0.3a	0.3a
Total	3.4			54.2	4.7	0.1

Strawberry tissues were sampled before (Time 0) and after fungicide treatments (Times 1 and 2). Mean values that are followed by the same letter within a column are not significantly different (*P* < 0.05).

**Figure 2:**
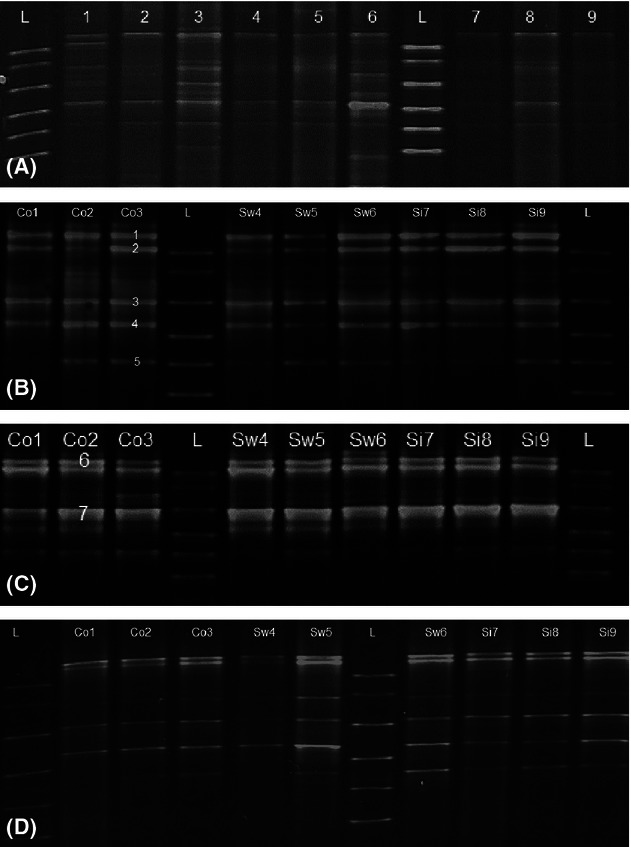
Field experiment. Denaturing gradient gel electrophoresis (DGGE) profiles of yeast communities on strawberry leaves and fruit at three sampling times, before (Time 0) and after treatment with fungicides (either Switch [Sw] or Signum [Si]) (Times 1 and 2). Each lane represents a composite sample of 10 plants per plot (the profiles of 3 of 4 plots are shown). Leaves sampled before treatments at Time 0 (A), unripe fruit sampled after treatment at Time 1 (B), and leaves and ripe fruit sampled after treatment at Time 2 (C and D, respectively). Untreated strawberry plants served as control (Co). L = DGGE ladder. Profile bands 1, 2, 3, 4, 5, 6, and 7 were excised and sequenced for identification of the respective yeasts (Table 3).

**Figure 3:**
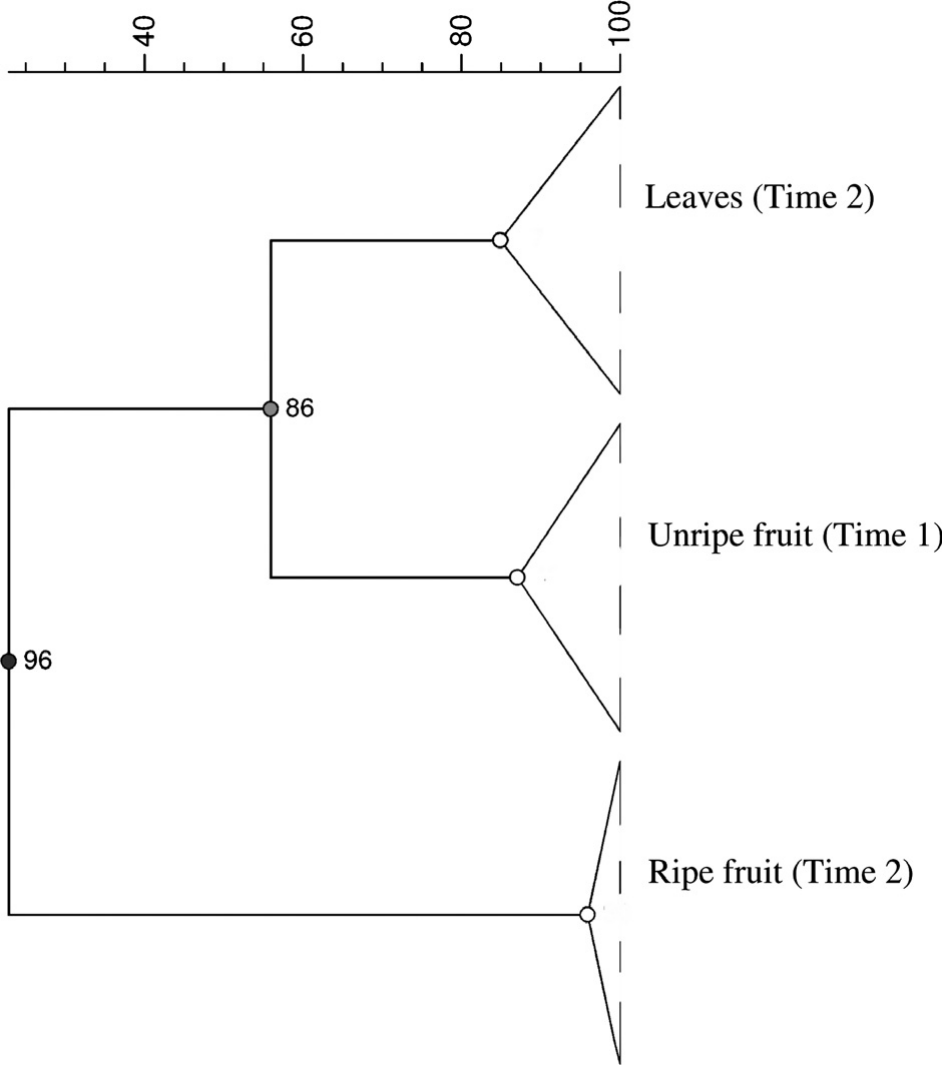
Field experiment. Relatedness of the denaturing gradient gel electrophoresis (DGGE) profiles of yeasts on leaves and fruits either treated or not treated with a fungicide (Switch or Signum) and from two sampling times (Times 1 and 2). The dendrogram was generated via band-based cluster analysis (UPGMA). Numbers on nodes are cophenetic correlations. Profiles clustered based on tissue type (leaf, unripe fruit, ripe fruit) and sampling time (Times 1 and 2), irrespective of fungicide treatment or fungicide type.

PCR-DGGE profiles at Time 0 were slightly heterogeneous (Fig. 2A). In contrast, at Times 1 (Fig. 2B, unripe fruit) and 2 (Fig. 2C, leaves; and Fig. 2D, ripe fruit), the profiles of each sampling set were similar between repetitions and treatments, with three to five main bands and two to three weak bands. Cluster analysis did not allow a clear distinction between the fingerprints generated from treated and untreated plants (similarity of the profiles was >85%, Fig. 3). In contrast, unripe fruit (Time 1), ripe fruit (Time 2), and leaves (Time 2) harbored significantly different yeast communities. Specifically, while the leaf profiles of samples taken at Time 2 still share 56% similarity with the DGGE profiles of unripe fruit sampled at Time 1, the DGGE profiles of the ripe fruit taken at Time 2 group separately with only 23% similarity (Fig. 3).

Seven dominant DGGE bands were extracted from gel (1 to 7, Fig. 2B and C), cloned, and sequenced. Tentative phylogenetic affiliations and GenBank accession numbers are presented in Table 3. Bands 1, 3, 5, 6, and 7 were associated with sequences of *Sporobolomyces*, *Cryptococcus*, and *Rhodotorula* yeasts. Multiple alignment confirmed that sequences of bands 1 and 6, and bands 3 and 7 were highly similar (97% and 99%, respectively). In addition, band 2 was associated with the yeast-like fungus *Aureobasidium pullulans* and band 4 had a high sequence homology with an isolate of *Cladosporium*. The detection of *Cladosporium* with the DGGE primers NL1-LS2 described for yeast was confirmed with a morphologically identified *Cladosporium* isolate. This isolate indeed produced a fragment in DGGE that comigrated with band 4 (data not presented). In addition, the five yeasts isolated on PDA plates (F1–F5, Table 1) were also analyzed in DGGE. Only the DGGE band from the *Rhodotorula* strain (F3, Table 1) comigrated with band 5 in the DGGE profiles, confirming the *Rhodotorula* identification of this band by DGGE band sequencing (Table 3). The other dominant culture types (F1, F2, F4, and F5) were not detected with the DGGE method (data not presented).

**Table 3:** Tentative phylogenetic affiliation of dominant epiphytic yeast types from strawberry leaves or fruit based on denaturing gradient gel electrophoresis (DGGE) band sequencing

				Closest sequence relatives		
				
Dominant DGGE band	Genbank accession #	Experiment location	Strawberry tissue	Strain(s)	Genbank accession #	Homology %
1	JN636797	field	unripe fruit	*Sporobolomyces roseus* ESAB18	AJ749836	100
2	JN636798	field	unripe fruit	*Aureobasidium pullulans* K-464	HE572524	100
3	JN636799	field	unripe fruit	*Cryptococcus victoriae*	FN667851	99
4	JN636801	field	unripe fruit	*Cladosporium bruhnei* CPC 5101	GU214408	99
5	JN636800	field	unripe fruit	*Rhodotorula graminis* WP1	EU563930	100
6	JN636802	field	leaf	*Sporobolomyces phaffii* AS 2.2137	AY070011	96
7	JN636803	field	leaf	*Cryptococcus victoriae* TSN-84	FR716586	100

### In vitro sensitivity of yeasts and fruit rot fungi to Switch and Signum

**Figure 4:**
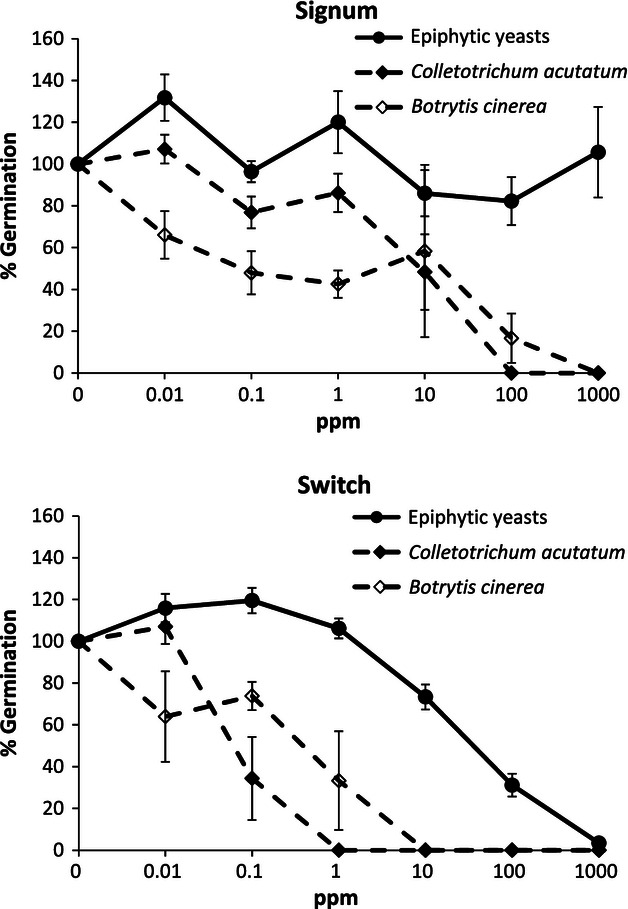
In vitro experiment. Mean germination (%) of epiphytic yeasts (5 isolates), *Colletotrichum acutatum* (3 isolates), and *Botrytis cinerea* (2 isolates) on potato dextrose agar amended with various concentration (ppm) of Switch and Signum.

Differences in germination between the epiphytic yeasts and the fruit rot fungi *B. cinerea* and *C. acutatum* were observed on Signum- and Switch-amended PDA plates (Fig. 4). All tested isolates were significantly more sensitive to Switch than to Signum. The epiphytic yeast isolates were significantly less sensitive to both fungicides as compared with the fruit rot fungi.

The sensitivity of the epiphytic yeast isolates to Signum was variable, with G5 and G2 significantly more sensitive than F4 and F5; F1 was intermediate sensitive. In contrast, there were no significant differences in the sensitivity of the epiphytic yeast isolates to Switch nor of the fruit rot isolates to both fungicides.

## Discussion

This is the first study investigating the effect of fungicides on epiphytic yeasts of strawberry. In vitro tests showed that epiphytic yeasts isolated from strawberry were at least 10 times less sensitive to Switch and Signum as compared with *C. acutatum* and *B. cinerea*, two important fungal strawberry pathogens and the targets for the fungicide control. Moreover, a greenhouse and field experiment using conventional plating and PCR-DGGE analysis showed that the density and diversity of the main epiphytic yeast species associated with strawberry were not affected by standard applications of Switch and Signum. Switch and Signum are two commonly used fungicides containing the active ingredients “cyprodinil plus fludioxonil” and “boscalid plus pyraclostrobin” and both fungicides reduced the incidence of strawberry fruit rot caused by *C. acutatum* and *B. cinerea* in the field.

Previous studies concerning the effect of fungicides on epiphytic yeasts were mainly done on grapes and grasses using conventional plating techniques. For example, Comitini and Ciani (2008) showed that standard fungicide treatment procedures on grapes (including “cyprodinil plus fludioxonil”) resulted in a dramatic reduction in yeast density and a shift in yeast communities. Similar research on grasses showed that equal or reduced amounts of yeasts were detected in the phyllosphere of fungicide-treated grasses as compared with the untreated control (Buck and Burpee 2002). These findings are in sharp contrast to the results of Cadez et al. (2010), showing that larger yeast counts were found on grapes treated with commonly used fungicides (iprodione, pyrimethanil, and “cyprodinil plus fludioxonil”) than on control samples. In sum, it is difficult to compare results from different studies due to different hosts, sampling strategies, washing procedure, and culture medium (Fonseca and Inácio 2006).

This study combined the performance of DGGE with cultural isolation on PDA for the analysis of yeast dynamics on strawberry plants. The dominant yeasts detected in DGGE seemed to be minor or nongrowers on the culture medium used. The reverse was also observed: only one yeast isolate (isolate F3, *Rhodotorula* sp.) detected on a culture plate could be attributed to a represented dominant strain in DGGE. Several factors may contribute to this low correspondence between the PCR-DGGE analysis and plating. First, culture-independent strategies such as PCR-DGGE have the advantage of detecting the occurrence of viable but nonculturable species (Muyzer and Smalla 1998; Giraffa and Neviani 2001). It has been suggested that in natural habitats, as much as 90–99% of the microflora may be viable but not culturable by agar plating (Amann et al. 1995; Ercolini et al. 2001; Vartoukian et al. 2010), and evidence of viable but nonculturable yeasts exists (e.g., Divol and Lonvaud-Funel 2005; Serpaggi et al. 2012).

Second, only one type of agar medium (PDA) was used for plating in this study. The type of growth medium used can greatly influence the growth rate/interaction and, by extension, the density and diversity of the yeast species isolated. Not one medium is optimal for the isolation of phylloplane yeast (Fonseca and Inácio 2006). Therefore, further research is needed to find out whether other agar media may reveal another range of phylloplane yeast species of strawberry that correspond to the dominant types revealed through DGGE.

Third, PCR-DGGE analysis does not seem to consistently discriminate between filamentous fungi, yeast-like fungi, and yeasts. Specifically, in this study the filamentous fungus *Cladosporium* and the yeast-like fungus or dimorphic species *Aureobasidium pullulans* cross-reacted with the DGGE primers described for yeasts. Similarly, in the study of Prakitchaiwattana et al. (2004), the fungal species *Phialocephala* and *Raciborskiomyces* on grapes were also cross-reacted using the same PCR-DGGE technique. Remarkably, although *Cladosporium* is a filamentous fungus and *Aureobasidium pullulans* is a yeast-like fungus, their presence was not affected by the application of the two fungicides. This *Cladosporium* strain is native for strawberry and may also be tested as potential candidate for biological control of strawberry fruit rot diseases combined with fungicide applications. Kohl et al. (2009) showed the potential of a *Cladosporium cladosporioides* strain in the control of apple scab.

Examination of the dynamics of the yeast communities on strawberry revealed differences in diversity and density depending on the growing systems (greenhouse vs. field conditions), strawberry tissue type (leaf, unripe fruit vs. ripe fruit), and sampling times. The effect of the growing system was demonstrated with different yeasts species and DGGE patterns on greenhouse- and field-grown strawberry leaves.

The effect of sampling time was already demonstrated in the greenhouse experiment, as shown by the dominance of extra DGGE bands at Time 1 as compared with Time 0. In the field experiment, the epiphytic yeast communities in the different pooled samples showed heterogenic DGGE profiles (Time 0) with less pronounced dominance of specific yeast types, which indicates the presence of transient yeast communities. On plants that had been present longer in the field (Times 1 and 2), the yeast communities evolved to more similar and thus stable communities, with only a few yeast types predominating. Smalla et al. (2001) observed a similar shift in the bacterial community present in the rhizosphere of strawberry. They observed a pronounced difference between the 16S rDNA patterns of the first sampling time as compared with the patterns of the following sampling times during the growing season. The effect of sampling time was also demonstrated in the plating results, showing a 93% reduction in the amount of yeasts on leaves at Time 2 (after spraying) as compared with Time 0 (before spraying). This reduction may be attributed to washing off the yeast by the spraying. More research is needed to elucidate the effect of sampling time/spraying events on the dynamic of epiphytic yeasts on strawberry.

The effect of tissue type on the yeast community was shown both in the DGGE and the plating analysis. First, when ripe fruit and leaves were sampled at the same time (Time 2), the yeast DGGE profiles were significantly different, indicating that ripe fruit is a very different substrate for the yeasts as compared to the leaves. Second, in the plating test, it was demonstrated that significantly more yeast colonies were counted on unripe fruit than on other strawberry tissues. It has been shown that the colonization of unripe fruit is an important step in the epidemiology of the fruit rot pathogens *B. cinerea* and *C. acutatum* (Bristow et al. 1986; Van Hemelrijck et al. 2010; Guidarelli et al. 2011). Our results suggest that unripe fruit could be a suitable target for protection with an artificial, uniform yeast coating. Note, however, that the high yeast concentration on unripe fruit could also be attributed to the effect of the sampling time, such as the day period and the weather conditions at that time point. The adhesiveness of epiphytic yeast is dynamic, with nonadherent cells making up a larger percentage of the community in the morning than in the afternoon (Allen et al. 2006). Therefore, more research is needed to elucidate the importance of plant tissue versus sampling time on the density of yeast species on strawberry.

Many epiphytic microbial ecology studies focus or include yeast species on plants (review: Fonseca and Inácio 2006), but knowledge about yeast ecology on strawberry is poor. All yeast species isolated in the present study were white or pink, which is typical for epiphytic yeasts in temperate zones (Buck and Burpee 2002). Specifically, the following yeast species were identified: *Candida*, *Pichia*, and *Metschnikowia* (ascomycetes) and *Cryptococcus*, *Sakaguchia*, *Sporidiobolus*, and *Rhodotorula* (basidiomycetes). Several strains belonging to these species have been described as biological control agents against important strawberry fruit rot pathogens (e.g., Lima et al. 1997; Helbig 2002; Wszelaki and Mitcham 2003; Karabulut et al. 2004; Mo and Sung 2005; Zhang et al. 2007, 2010; Huang et al. 2011). The isolated *Rhodotorula* sp. strain F3 deserves special attention, as this was the only strain detected with both the plating and the PCR-DGGE technique.

In conclusion, this study demonstrates that (i) dominant yeasts on strawberry leaves and fruit are less sensitive to two commonly used fungicides as compared with the target organisms, *B. cinerea* and *C. acutatum*, and (ii) the application of these two fungicides does not affect the diversity of the epiphytic yeast community in the greenhouse or field. In addition, the epiphytic yeast communities evolve during growth of the plant and maturation of the fruit. This study opens avenues for further research. One example is research on the biocontrol activity of yeasts that are strongly represented on leaves and fruit for which we now have isolates for testing, such as *Cryptococcus*, *Sporobolomyces*, and *Rhodotorula*.
